# Which patients and orthopedic material do get infected with Gram-negative non-fermenting rods?

**DOI:** 10.1186/2047-2994-4-S1-P72

**Published:** 2015-06-16

**Authors:** I Uckay, C Landelle, A Agostinho, M Al-Mayahi, D Pittet

**Affiliations:** 1 Geneva University Hospitals, Geneva, Switzerland; 2 Infection Control Program, Geneva University Hospitals, Geneva, Switzerland; 3 Infection Control Program, Geneva University Hospitals, Geneva, Switzerland

## Introduction

The 1^st^ and 2^nd^ generation cephalosporins used for perioperative prophylaxis do not cover non-fermenting Gram-negative rods (NFR).

## Objectives

We investigated patient populations and types of surgery at risk for Gram-negative infections overall, and NFR in particular.

## Methods

Retrospective cohort study of adult patients operated for orthopedic infections between 2004 and 2014. Only the first episode of infection was considered for analysis and infection diagnosis was based on intraoperative samples.

## Results

The median age of all patients was 57 years; 871 were females, and 1021 were immune-suppressed. Overall, 665 episodes (24%) involved osteosynthesis material (321 arthroplasties, 150 plates, 54 nails, and other materials). The median duration of antibiotic prescription prior to intraoperative sampling was 4 days; it occurred in 42% of all cases. Of the total 2740 surgical procedures, 568 grew Gram-negative pathogens (21%) of which 258 (9%) were NFR (120 episodes as co-infection) and 178 (7%) *Pseudomonas aeruginosa*. Prior antibiotic use was significantly associated with NFR infections (159/947 vs. 99/1478; *p*<0.01). On the median, NFR patients yielded 7 days of prior antibiotic use compared to 3 days of patients with non-NFR infections (*p*<0.0001). Additional conditions associated with NFR infections were the presence of plates (25/144 vs. 233/2281; *p*<0.01) the presence of diabetic foot (57/385 vs. 201/2040; *p*<0.01). Besides plate infection, no other hardware or prosthesis was particularly involved in NFR infections. Risk was associated with an age older than 80 years. In this age category, NFR were responsible for 11% of all pathogens among orthopedic infections. In contrast, NFR were almost never documented in septic bursitis and less frequently associated with abscess formation in native bone or prosthetic joint infections. NFR infection was also less frequently identified in shoulder infections (3/80 vs. 255/2345; *p*=0.04).

## Conclusion

The most important finding associated with orthopedic infections due to NFR is prior antibiotic use in elderly patients with diabetic foot infections/problems.

## Disclosure of interest

None declared.

